# Improving Signal and Photobleaching Characteristics of Temporal Focusing Microscopy with the Increase in Pulse Repetition Rate

**DOI:** 10.3390/mps2030065

**Published:** 2019-07-28

**Authors:** Viktoras Lisicovas, Bala Murali Krishna Mariserla, Chakradhar Sahoo, Reuben T. Harding, Michael K. L. Man, E Laine Wong, Julien Madéo, Keshav M. Dani

**Affiliations:** 1 Femtosecond Spectroscopy Unit, Okinawa Institute of Science and Technology Graduate University, 1919-1 Tancha, Onna-son, Okinawa 904-0495, Japan; 2 Department of Physics, Indian Institute of Technology, Jodhpur, Rajasthan 342037, India

**Keywords:** two-photon microscopy, wide-field excitation, live imaging, temporal focusing

## Abstract

Wide-field temporal focused (WF-TeFo) two-photon microscopy allows for the simultaneous imaging of a large planar area, with a potential order of magnitude enhancement in the speed of volumetric imaging. To date, low repetition rate laser sources with over half a millijoule per pulse have been required in order to provide the high peak power densities for effective two-photon excitation over the large area. However, this configuration suffers from reduced signal intensity due to the low repetition rate, saturation effects due to increased excitation fluences, as well as faster photobleaching of the fluorescence probe. In contrast, with the recent advent of high repetition rate, high pulse energy laser systems could potentially provide the advantages of high repetition rate systems that are seen in traditional two-photon microscopes, while minimizing the negatives of high fluences in WF-TeFo setups to date. Here, we use a 100 microjoule/high repetition rate (50–100 kHz) laser system to investigate the performance of a WF-TeFo two-photon microscope. While using micro-beads as a sample, we demonstrate a proportionate increase in signal intensity with repetition rate, at no added cost in photobleaching. By decreasing pulse intensity, via a corresponding increase in repetition rate to maintain fluorescence signal intensity, we find that the photobleaching rate is reduced by ~98.4%. We then image live *C. elegans* at a high repetition rate for 25 min. as a proof-of-principle. Lastly, we identify the steady state temperature increase as the limiting process in further increasing the repetition rate, and we estimate that repetition rate in the range between 0.5 and 5 MHz is ideal for live imaging with a simple theoretical model. With new generation low-cost fiber laser systems offering high pulse energy/high repetition rates in what is essentially a turn-key solution, we anticipate increased adoption of this microscopy technique by the neuroscience community.

## 1. Introduction

In recent years there is ever-increasing interest in the neuroscience community for obtaining access to a more holistic view of neural activity, which has motivated the development of newer bio-optical technologies. Two-photon laser scanning microscopy is one of the tools of choice often employed for this purpose. While it offers excellent resolution and is minimally invasive, it is also constrained by the speed at which scanning can be realized [1]. An extension of this technique using adaptive optics to generate multi-focal excitation is one way to increase the scan speed [2]. Another way is to employ the temporal focusing phenomenon, which offers a substantial increase in the area that can be accessed per single exposure cycle combined with useful axial sectioning characteristics [3].

Wide-field temporal focusing microscopy (WF-TeFo) is a far-field multiphoton imaging technique, which allows for excitation over a large area of the sample, which thus eliminates temporal drift in the lateral plane. It also could potentially enable a dramatic increase in acquisition rate for volumetric fluorescence imaging. In this scheme, ultrashort laser pulses are dispersed by a grating and focused again by an objective lens. This leads to a temporal focusing phenomenon, whereby the shortest pulse duration is achieved at the focal plane, and a rapid broadening is observed away from focus [4,5]. The gain in optical sectioning from a combination of quadratic excitation dependence on intensity and confinement that is offered by temporal focusing allows for the use of a weakly focused excitation spot with long lateral diameter, yet a narrow axial spread. An entire imaging plane can be illuminated at once with minimal out-of-focus fluorescence background. Information from all pixels can be gathered in parallel, since the whole image plane need not be scanned sequentially.

The efficient excitation of large areas with WF-TeFo requires high pulse energies, since excitation is spread out in a larger area than conventional point-scanning approach. Until recently, the laser systems that would be capable of delivering the required pulse energies at wavelengths that are compatible with two-photon cross sections of neural activity probes could only do so at relatively low repetition rates (~1 kHz). In the absence of control over repetition rate, one would rely on increasing the pulse energy to increase the fluorescent signal intensity. However, this has the undesired consequences of increased photo-induced damage, and furthermore is fundamentally limited by the saturation of fluorophore molecules. The new generation of fiber laser systems offers increased and fully adjustable repetition rates without sacrificing energy per pulse [6]. However, the repetition rate is limited by thermal considerations. Exposing tissue preparations or live samples to high average power illumination leads to temperature build-up, which is detrimental to live tissues [7]. In principle, an optimal combination can thus be fine-tuned by appropriately choosing the intensity and repetition rate, and other parameters until the desired experimental conditions are achieved [8].

In this work, we demonstrate large improvements in the strength and persistence of fluorescence signal by varying excitation laser repetition rate and peak power density applied to volumetric imaging of micro-bead samples and validate this result with neural activity recording of live *C. elegans*. We argue that the increase in repetition rate within the thermal limits is extremely beneficial for WF-TeFo two-photon imaging applications.

## 2. Materials and Methods

### 2.1. Temporal Focusing Microscopy Setup

**Figure 1:**
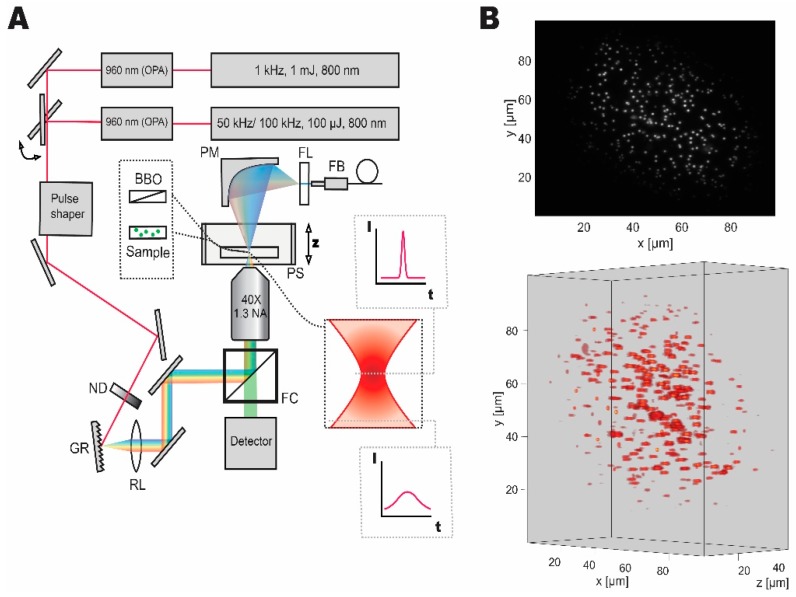
Dual laser setup for temporal focusing with pulse control and characterization capabilities. (**A**) Wide-field temporal focusing microscopy setup comprised of a grating (GR), relay lens (RL), and an objective lens was coupled to two laser systems with different repetition rates, that can be switched between with a flip mirror. Imaging samples were mounted on a high-speed piezo stage (PS). Discrete neutral density filter wheel (ND) was used to control the average power of optical excitation on the sample. During imaging experiments, the filter cube (FC) was used to separate the fluorescent signal for detection. For pulse shape characterization the second harmonic signal from a nonlinear crystal (BBO) mounted on a microscope slide was collected by a parabolic mirror (PM) passed through a filter (FL) and coupled to the fiber (FB) spectrometer of the pulse shaper for characterization and compression. (**B**) Grayscale two-dimensional and volumetric reconstructions of the fluorescent micro-bead sample demonstrating the Gaussian distribution of excitation light and accessible volume and resolution.

Our temporal focusing microscope setup was designed and implemented based on the published reports [4,9,10]. Figure 1 shows the schematic diagram of the setup. The first laser system, a low repetition rate, high pulse energy laser (Spitfire, Spectra-Physics, Santa Clara, CA, United States) was set to yield 140 fs, 1 mJ pulses at 1 kHz repetition rate, and 800 nm central wavelength. An optical parametric amplifier (Topas, Light Conversion, Vilnius, Lithuania) was used to obtain pulses at 960 nm wavelength (optimal wavelength for employed fluorescent probes). The second laser system, which is a high-repetition-rate source (Wyvern, KMLabs, Boulder, CO, United States), was set to either 50 kHz (for fluorescent bead experiments) or 100 kHz (for live imaging) providing 45 fs pulses with 100 µJ pulse energy at 800 nm central wavelength. A second optical parametric amplifier (TopasHR, Light Conversion, Vilnius, Lithuania) was used to convert the laser output to 960 nm central wavelength. Switching between the two laser sources was implemented with a flip mirror.

A pulse shaper (MIIPS Box 640, Biophotonics Solutions, Marlborough, MA, United States) with an integrated spectrometer was used to measure spectrum, as well as to characterize and compress the pulses to transform limited pulse duration. A neutral density filter wheel (ND 0.04 to 4, Thorlabs, Newton, NJ, United States) was used to control the average power after the pulse shaper. The beam was then directed to the temporal focusing microscopy setup, consisting of a reflective diffraction grating (1200 lines·mm^−1^, Thorlabs, Newton, NJ, United States), a relay lens (2 inches, 500 mm focal length, Newport Corporation, Irvine, CA, United States), and a microscope objective (UPLFLN 40XO, Olympus, Tokyo, Japan). We used a stripped down inverted microscope (IX71, Olympus, Tokyo, Japan) as a base. Nanopositioning piezo stage (Nano-Z50HS, Mad City Labs Inc., Madison, WI, United States) was used to house and scan through samples in the axial direction.

The fluorescence signal was separated while using a filter cube consisting of a long-pass filter with a cut-off at 800 nm, a dichroic mirror reflecting the near-infrared and transmitting between 675 to 500 nm, and a short-pass filter with a cut-off at 575 nm. Thus, the filter cube reflects the infrared light and transmits the epifluorescent signal to the detector. The signal was then measured while using a CMOS camera (Zyla 5.5, Andor Technology, Belfast, United Kingdom). In all imaging experiments, we collected data from the region-of-interest on the detector. The scale of the image on the sensor was measured to be 6.2 pixel·µm^−1^ (using a 10 µm calibration sample, Olympus, Tokyo, Japan). Laser power was measured while using a microscope slide power meter (S175C, Thorlabs, Newton, NJ, United States). The comparison between the laser systems was made based on peak power density, which is defined as the power delivered by an ultrafast pulse at its peak, divided by area of the beam waist. It was calculated using Equation (1):

(1) $$PPD=\frac{P_{A}}{A\cdot f\cdot \tau },$$

where PPD is the peak power density, P_A_ is the measured average power, f is the repetition rate or a number of pulses per second, A is the area of the beam waist, and τ is the pulse duration. Here, a square pulse with duration τ approximates Gaussian temporal pulse, and equal power density within the beam waist is assumed. When characterizing the excitation pulses, a parabolic mirror (50 mm focal length) was used to collect the second harmonic signal that was then coupled to the fiber spectrometer of the pulse shaper. An absorption filter (575 nm short-pass, Newport Corporation) was used to isolate the second harmonic signal generated from a microscope slide mounted beta barium borate nonlinear crystal (BBO, EKSMA Optics, Vilnius, Lithuania). We have measured the pulse duration of 142 fs at the sample for 1 kHz source and 235 fs for 50/100 kHz source.

### 2.2. Imaging Samples

We used fluorescent microspheres (Fluoresbrite^®^ YG Microspheres, Calibration Grade 1.00 μm, Polysciences, Inc., Warrington, PA, United States) as a surrogate sample to approximate the green fluorescent protein (GFP) based fluorescent probes. We have characterized the lifetime (2.4 ns) and excitation/emission spectra using time-resolved spectroscopy (Streakscope, Hamamatsu Photonics, Hamamatsu, Japan) and found that it closely resembles that of GFP [11]. The beads were fixed in an aqueous polyacrylamide gel (Crosslinking Reagents and Catalysts 29:1, Bio-Rad, Hercules, CA, United States) that was sandwiched between a microscope slide and cover glass with a silicon isolator as a spacer for casting (Silicone Isolators™, Grace Bio-Labs, Bend, OR, United States). All of the imaging experiments on the beads were performed at 50 ms exposure time.

For live animal experiments, *C. elegans* were cultured using standard cultivation methods [12]. Strain LX2004 was used for imaging experiments (*lite-1(ce314)*; *vsIs183*; *lin-15(n765ts) X [nlp-3::GCaMP5G]*) [13]. The animals were physically immobilized in a microfluidic device that was modified from [10] in standard M9 minimal medium [14]. 1M glycerol solution in M9 medium was used as the stimulation solution. All of the chemicals used in this work were purchased from Nacalai Tesque (Kyoto, Japan). Stimulation timing was controlled by a computer that was connected with a perfusion valve control system (VC-6, Warner Instruments, Hamden, CT, United States).

Data acquisition and analysis were performed while using custom-written Matlab (Mathworks, Natick, MA, United States) scripts. All *C. elegans* experiments were performed at 30 ms exposure time. Detector background was subtracted from raw measurements of signal intensity and fit while using Matlab’s built-in curve fitting toolbox. Confidence intervals were derived using non-parametric resampling methods, namely bootstrapping from Matlab’s statistical toolbox. Animal imaging data were analyzed using Fiji software [15]. Section data was processed by subtracting background, denoising, and converting into volumetric representation by stacking individual slices. Neuronal positions were then manually annotated, and the fluorescence signal was extracted over time.

## 3. Results

### 3.1. Signal Intensity Increases Linearly with the Repetition Rate

In fluorescence imaging, photons that were emitted by the fluorophores are the carriers of the location and density information. The acquisition rate is fundamentally limited by the number of photons that can be captured by a detector during the exposure time. This is especially critical in multiphoton microscopy, where excitation/emission processes are limited by power-law dependency on excitation intensity [16]. Multiphoton microscopy requires the use of pulsed excitation sources to satisfy the requirement for high excitation intensity. The number of emitted photons can be increased by ramping up the intensity and the pulse repetition rate [8]. Increasing pulse intensity yields benefits up to a point where all of the available fluorophores have been saturated. On the other hand, increasing the repetition rate should produce gains in the signal, as long as the time between the pulses is shorter than the fluorophore lifetime.

**Figure 2:**
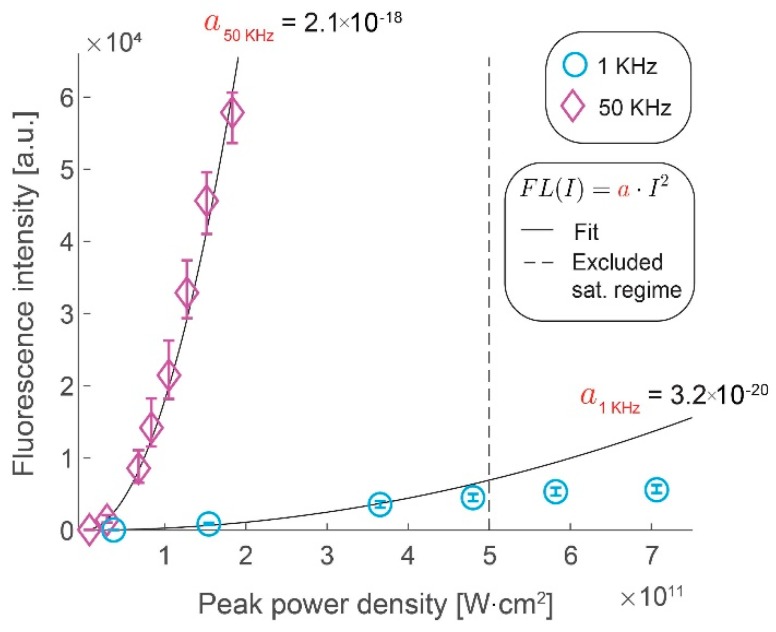
Gains in fluorescence intensity with increased pulse repetition rate. Fluorescence intensity increases with the peak power density of excitation light shown for 1 kHz (circles) and 50 kHz (diamonds). Quadratic fits with a single coefficient are shown as black lines (1 kHz, R^2^ = 0.97, 50 kHz, R^2^ = 0.99). Data points beyond a saturating peak power density value were excluded from the fit. Error bars represent bootstrapped confidence intervals for the mean fluorescence intensities calculated at α = 0.01 confidence level. Data derived from n = 117 (1 kHz) and n = 41 (50 kHz) measurements of fluorescent microspheres, within 10% of the area around the excitation beam maximum.

We assess the gains in the collected signal by imaging fluorescent microspheres by increasing the excitation pulse repetition rate from 1 kHz to 50 kHz. Figure 2 shows the gains in fluorescence intensity with increase in peak pulse intensity for the two repetition rates. We confirm that the former scales quadratically with the latter, as long as the saturation limit of the fluorophores has not been reached, by measuring the fluorescence intensity at different peak power densities. At higher peak power densities (above 5 × 10^11^ W⋅cm^−2^) at 1 kHz repetition rate, we clearly observe that saturation effects begin to take effect as the data deviates from this quadratic scaling. We demonstrate that the increase in the laser repetition rate results in a roughly proportional increase in the signal intensity by comparing the rate of quadratic rise of the fluorescence signal with the peak power density (i.e., a_50kHz_ = 2.1 × 10^−18^ W^−2^⋅cm^−4^ vs. a_1kHz_ = 3.2 × 10^−20^ W^−2^⋅cm^−4^). The observed saturation range is in line with the literature of conventional two-photon microscopy [17] and it illustrates a challenge in using the WF-TeFo excitation scheme. Until recently, the excitation intensities required to drive a two-photon process over wide-field excitation were only achievable from low repetition systems. However, increasing the excitation intensity yields no benefit past the saturation limit. Given improvements in laser technology, it would be much more beneficial to increase the repetition rate. It is especially true for studying biological processes that occur on short timescales, such that longer integration times are not practical.

### 3.2. Photobleaching Rate Increases with Peak Intensity, but Not with the Repetition Rate

We sought to test how the increase in repetition rate affects the photobleaching rate after confirming that a higher fluorescence signal can be achieved with an increased pulse repetition rate. Taking photoinduced damage into account is essential for the implementation of any fluorescence-based experiment. Any commonly used fluorophore has only a limited number of excitation-decay cycles that can be undergone before the fluorophore is permanently depleted [18]. This process is accelerated when fluorescent molecules are exposed to elevated temperature or illumination intensity [19]. In particular, large excitation intensities result in severe degradation of fluorophores. Under these conditions, the transition to higher-order excited states is favored, and as a result, covalent bond cleavage and increased reactivity are promoted [18]. The severity of photoinduced damage non-linearly scales with illumination intensity [20]. Two-photon microscopy, in general, requires substantial excitation intensities due to the inherent inefficiency of the two-photon process [8]. In addition to this, the lateral profile of the excitation disk in WF-TeFo and the resulting fluorescence intensity follow a Gaussian distribution. It is sometimes desirable to ramp up excitation intensity to maximize the lateral excitation area within tolerance levels of photobleaching in the center.

**Figure 3:**
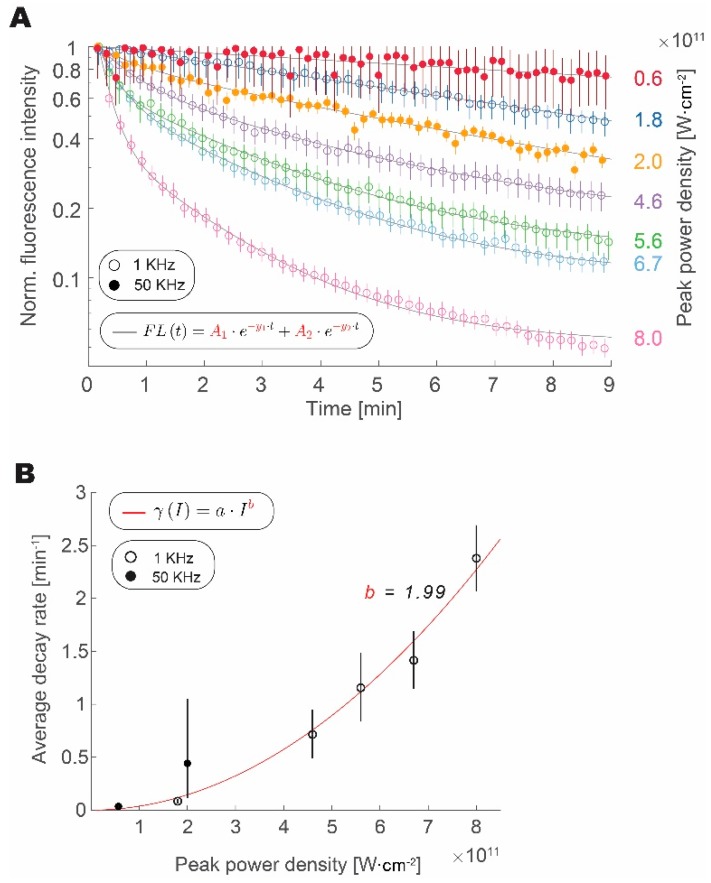
Increased repetition rate does not enhance photobleaching. (**A**) Loss of signal intensity over time with continuous excitation normalized to maximum and shown for 1 kHz (circles) and 50 kHz (full circles) at different peak intensities. (**B**) Amplitude weighted average decay rate scaling with pulse peak intensity for photobleaching at 1 kHz (empty circles) and 50 kHz (full circles) fitted with a power law function (R^2^ = 0.96). In all plots, error bars represent bootstrapped confidence intervals of the mean calculated at *p* = 0.05 confidence level. Data derived from *n* = 157–214 (1 kHz) and *n* = 32–56 (50 kHz) measurements of fluorescent microspheres, within 10% area around Gaussian excitation beam maximum.

We have measured the fluorescence from fluorescent microspheres over time at different intensities and repetition rates (1 or 50 kHz) to test how photobleaching rate scales with an increase in pulse peak intensity and repetition rate, as shown in Figure 3. We confirmed that the increase in peak intensity expedited the loss of fluorophore fluorescence, regardless of the repetition rate. Data from both 1 kHz and 50 kHz experiments align well with increasing peak pulse intensity. The bleaching traces are well approximated by a double exponential decay [20]. Amplitude weighted average of the decay follows a power law with a quadratic exponent. This result is similar to reports studying the photobleaching rate in GFP [21]. Data from the 1 kHz and 50 kHz groups follow this trend, thus indicating that increasing repetition rate has a much lower impact on photobleaching rate than the increase in peak power density. From fits derived in Figure 1 and Figure 2, we can calculate the ratio of average decay rates for the peak power densities that are required to obtain the same fluorescence intensity:

(2) $$\frac{\gamma_{50KHz}}{\gamma_{1KHz}}={\left(\frac{I_{50KHz}}{I_{1KHZ}}\right)}^{\frac{1}{2}\cdot b}={\left(\frac{\alpha_{1KHz}}{\alpha_{50KHZ}}\right)}^{\frac{1}{2}\cdot b}≃\frac{\alpha_{1KHz}}{\alpha_{50KHZ}}$$

In our experiments, we calculated a 98.4% decrease in the average photobleaching rate that was obtained by increasing the repetition rate and decreasing the peak power density, while maintaining a constant signal intensity.

### 3.3. Temperature Constraints Indicate an Optimum Repetition Rate Between 0.5 and 5MHz

Heating resulting from illumination of biological tissues with high-intensity laser light results in adverse effects, even at levels that are much lower than would be required to induce structural damage. Small increases in temperature induce changes in neural activation patterns and cellular metabolism [7]. Recently, the modeling of temperature dissipation in two-photon excitation has received considerable attention. Rigorous modeling of the increase in temperature while using Monte Carlo simulations [7,22], as well as deriving analytical solutions from three-dimensional (3D) Fourier equation using Green’s functions [23] has been reported and verified by experiments.

We wanted to estimate how much we can increase the pulse repetition rate before thermal load becomes too high for biological samples after ascertaining that an increased pulse repetition rate leads to improvements in fluorescence signal intensity and shows no increase in photobleaching. The highest repetition rate system that was capable of driving WF-TeFo in wide-field excitation at our disposal was limited to 100 kHz. Under these conditions, we did not observe any apparent thermal damage. Therefore, we set out to estimate the limits for how much average power can be safely applied to a biological sample using WF-TeFo through modeling.

In this work, we have opted to use a simplified first approximation model that relies on steady-state temperature dissipation in idealized brain tissue labeled with a GFP based probe. It is based on ideas that were put forward to estimate heat accumulation in point scanning two-photon microscopy [8]. In WF-TeFo, the combination of spatiotemporal dispersion, loose-focusing, and high numerical aperture results in a beam that is rapidly converging towards and away from the focal plane [9]. As the light propagates through tissue, it is attenuated by scattering and absorption [24]. The highest intensities of the excitation light are achieved in the focal region and they reduce rapidly outside the Rayleigh length.

To simplify the problem, we define the excitation spot as a cylinder with uniform intensity distribution, the radius of which is limited by the full-width-half-max of lateral intensity distribution and height is constrained by the Rayleigh range. The heat that accumulates in this region due to absorption can dissipate into the surrounding tissue laterally, but it is restricted by the geometry of the beam to do so in the axial direction. We further assume that the photons absorbed by the tissue are dissipated as heat or are converted to two-photon emission and that the contributions arising from higher-order absorption of the tissue are negligible. When both one and two-photon absorption contributions are taken into account, the fraction of absorbed photons in a Gaussian pulse can be expressed as (variable definitions are provided in Table 1):

(3) $$F_{a}=\frac{N_{a}}{N_{\tau }}=\frac{N_{1\lambda }+(N_{2\lambda }-N_{em})}{N_{\tau }}=\left(1-e^{-\mu_{a}\cdot l}\right)+\left(I\cdot S\cdot \delta_{2\lambda }\cdot C\cdot l\cdot \frac{\lambda }{hc}\right)\cdot \left(1-\frac{\Phi }{2\sqrt{2}}\right).$$

This expression is formulated assuming that the fluorophore lifetime is shorter than the interval between two consecutive excitation pulses, and all of the fluorophores have sufficient time to go back to the ground state during this time.

We can assume that there is negligible dissipation of heat due to convection and radiation and the primary mode of heat transfer is by conduction since we are evaluating imaging in a tissue composed infinitesimally small cells. Additionally, metabolic heat and blood circulation are neglected in this analysis. We model the focal volume as a cylinder with a uniform heat source surrounded by an infinite heat sink. We begin by defining a source term:

(4) $$\varepsilon =\frac{P_{A}\cdot F_{a}}{V}.$$

The heat conduction balance from the center through the outer shell at some distance *r* to *r* + Δ*r* can be described as:

(5) $${\left|2\pi rl\cdot q_{r}\right|}_{r}-{\left|2\pi rl\cdot q_{r}\right|}_{r+\Delta r}+2\pi r\cdot \Delta r\cdot l\cdot \varepsilon =0.$$

We solve for *q_r_* by taking a limit Δ*r* → 0 and integrating the resulting expression to obtain:

(6) $$q_{r}=\frac{\varepsilon \cdot r}{2},$$

taking into account that *q_r_* is finite at *r* = 0. Using Fourier’s law, the thermodynamic heat flow balance at some *r* can be expressed as:

(7) $$-k\frac{dT}{dr}=\frac{\varepsilon \cdot r}{2}.$$

Given the assumption of infinite thermal sink outside the cylinder (*T* = *T*_0_, when *r* = *R*), integration of Equation (7) yields:

(8) $$\Delta T=\frac{\varepsilon \cdot R^{2}}{4\cdot k}.$$

**Figure 4:**
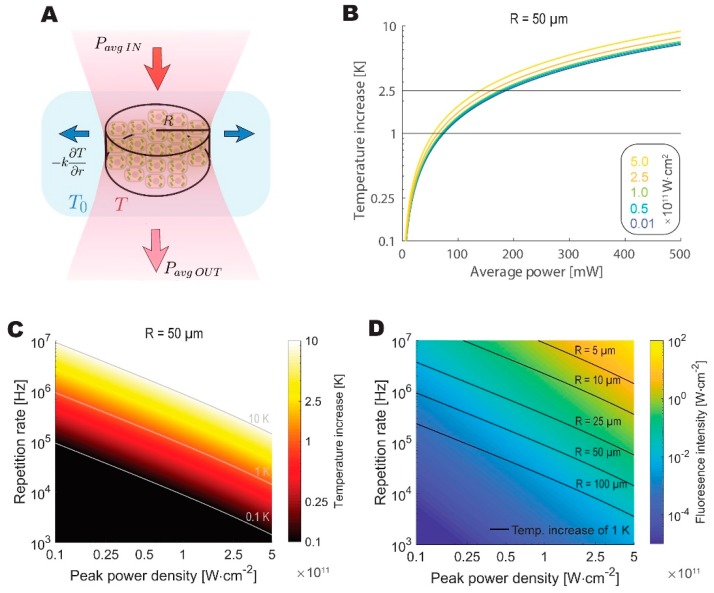
The theoretical analysis highlights thermal constraints on feasible repetition rates for live imaging. (**A**) Schematic depiction of a thermodynamic equilibrium reached between the absorbed fraction of average excitation power and lateral dissipation by the tissue. Beam waist is modeled as a cylinder of varying radius. (**B**) Increase in temperature at the sample plotted against average power for beams with different peak power densities for 50 µm radius spot. (**C**) The temperature increases as a function of peak power densities and repetition rates calculated for a 50 µm radius spot. (**D**) Calculated fluorescence intensity as a function of peak power densities and repetition rates. Isothermal lines denote the combination of parameters, where a 1 K increase in temperature is reached for excitation cylinder of different radii.

Figure 4 presents the modeling results. Our model predicts the maximum average power that can be safely applied to the biological sample at around 100 mW, which is close to the limit that was determined by Podgorski and Ranganathan [7]. To draw a further comparison with the values reported by Podgorski and Ranganathan, we have calculated the predicted increase in temperature given 200 mW of average power, 80 MHz repetition rate, 7 µm beam spot radius, and 250 µm imaging depth, obtaining a nearly identical temperature increase of 6.2 K. Despite its simplicity, the model is in good agreement with a significantly more rigorous simulation.

We then looked at a different repetition rate and peak power density combinations to identify the optimal regime for WF-TeFo, given thermal considerations. The optimal repetition rate and power density combination are constrained by fluorescence signal intensity and photobleaching as well as the thermal dissipation, which is dependent on the radius of the excitation spot. When these considerations are taken into account, the optimal pulse repetition rate lands in the range between approximately 0.5 MHz and 5MHz for the imaging spot diameters that were reported in the literature and used in this work (25 to 100 µm, and assuming excitation peak power densities at least an order of magnitude below saturation).

### 3.4. Live Sample Imaging at 100 kHz Yields High Signal Intensities without a Photomultiplier

We pushed our high repetition system to its maximum of 100 kHz as a proof-of-principle to verify our findings. We employed the live imaging of *C. elegans* expressing GCaMP5G calcium indicator in a subset of aversive neurons (including ASH aversive neuron pair) to evaluate the performance of our imaging system. GCaMP5G is an enhanced derivative of GFP with engineered proteins domains for sensing calcium concentration in vivo [11,32]. Therefore, we expected to observe comparable fluorescence signal improvements as in experiments while using fluorescent microspheres under similar excitation conditions.

**Figure 5:**
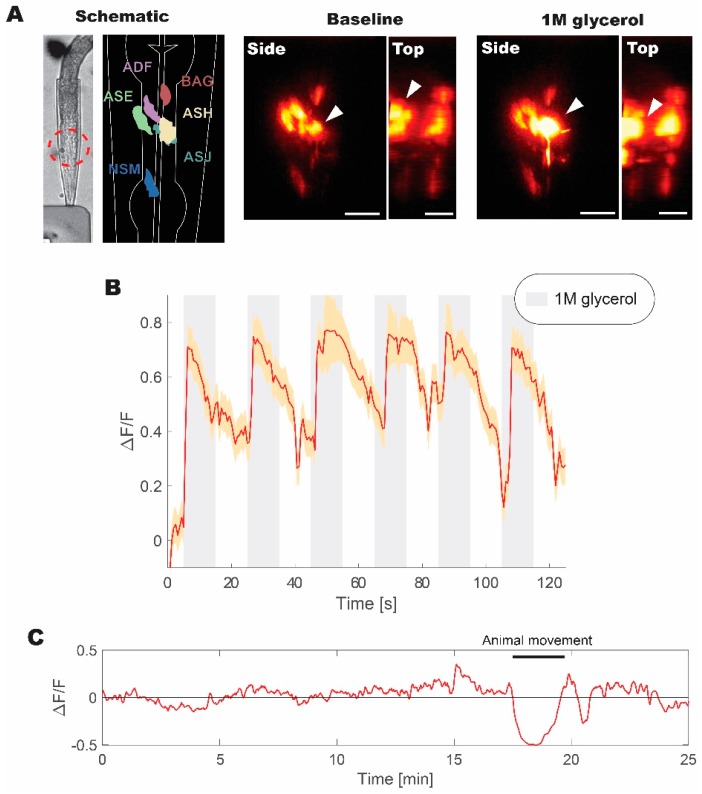
Live volumetric imaging of *C. elegans* responding to 1 M glycerol solution measured at 100 kHz. (**A**) Bright-field image of animal immobilized in a microfluidic device, and corresponding schematic diagram of imaged neurons, as well as examples of neuronal calcium activity at the baseline and when stimulated with 1M glycerol solution (shown from the side and top directions). Red dashed circle indicates the imaged region of interest. White arrows indicate the position of ASH sensory neuron cell body. (**B**) Normalized fluorescence change recorded for six pulses of 1M glycerol with a period of 40 s between pulses. (**C**) Continuous volumetric recording of baseline neural activity in ASH neuron without stimulus application. A sudden decrease in the signal at 18 min. is due to animal movement in the microfluidics device. Volumetric data were acquired at ~2 Hz with 30 ms exposure per slice. Normalized fluorescence change was calculated from the mean of volumetric fluorescence. Error bars represent standard error of the mean derived from measurements of individual pixels in the recording. Scale bars denote 10 µm.

The animal was trapped in a microfluidics device and was repeatedly stimulated with 1M glycerol to induce aversive responses [33]. In this configuration, we were able to obtain volumetric recordings over the volume of the head (Figure 5). A subset of head neurons expressing fluorescent probe was visible under WF-TeFo excitation when only using a CMOS camera with 60% QE without a photo-multiplication device. This was not possible in our experiments using a 1 kHz source and, as reported by others, using 10 kHz laser setup [10]. Additionally, we were able to extract fluorescence change (normalized to the baseline) showing reliable responses to the presentation of 1 M glycerol stimulus. The WF-TeFo imaging system in this configuration offers sufficient resolution to see axons, while maintaining a large field of view and without the necessity to use photomultiplier devices. Finally, we tested the applicability of our system for continuous imaging of neural activity. We ascertained that over 25 min. of continuous imaging without stimulation, the baseline fluorescence has essentially remained stable (note that observed dip in the signal in Figure 5C is due to animal motion).

## 4. Discussion

In this work, we have assessed the advantages that are offered by high repetition laser sources for WF-TeFo. We have demonstrated that a moderate increase in repetition rate does not adversely affect photobleaching characteristics, but rather significantly enhances the fluorescence signal. Increasing the repetition rate, in our hands, allowed for the implementation of a setup while solely using off-the-shelf components [34]. Nevertheless, this setup was capable of acquiring clear readouts of neuronal activity from live *C. elegans* animals under aversive stimulation. The signal intensity change was so substantial that calcium signals could be qualitatively inferred from reconstructed volumes, without reliance on quantitative analysis. Others have also indicated the benefits of increased repetition rate on temporal focusing, for a moderate area [35] at 250 kHz and smaller area size [36] at 4–5 MHz combined with scanning. We expand on these observations by also including the measures for photobleaching and estimating the upper limits of pulse repetition rates that can be safely employed. When considering that higher repetition rates could be used to increase the signal intensity, it is possible to significantly speed up the rate of volumetric imaging by reducing the exposure time. As such, WF-TeFo with high repetition sources opens new opportunities for investigating the flow of information in neural networks. However, high-power laser amplifier systems, like ‘Spitfire’ and ‘Wyvern’, are not commonly available to biologists.

Since the multiphoton microscopy revolution in the early 90s, the excitation source has been the primary consideration and a major expense for implementation of two-photon imaging solutions [8]. Despite expectations, the situation has not changed much since then [16]. Until recently, researchers focusing on bio-imaging were mostly limited to the use of solid-state laser oscillator systems that are based on Kerr-lens mode locking in titanium-doped sapphire (Ti: Sa) crystal. These systems are readily available from commercial vendors as a turn-key solution and they offer automated wavelength tunability; however, in most cases, they lack sufficient pulse energies for WF-TeFo. On the other hand, the added control of repetition rate, while maintaining high pulse energy, necessitates the use of regenerative amplifiers, which are complex optical systems that require expertise in managing and operating.

Recent advances in fiber lasers promise much more compact laser sources with full control of repetition rate in a reliable and easy to operate package. Until recently, the wavelength tunability was the only limitation [6]. Advances in non-linear optical parametric amplifiers that come attached to high power/high repetition rate laser systems (i.e., Spirit and Spirit-NOPA, Spectra-Physics ^®^) offer tunability in wavelength and repetition rate without the need to tinker with laser optics after deployment, and, at the same time, offers high pulse stability. These systems are still expensive, but we expect that as they become *de facto* sources for multiphoton imaging, the prices will decrease. We predict that the new generation of fiber lasers will generate renewed and growing interest in temporal focusing technology, given the advantages in speed and simplicity that are offered by WF-TeFo over point scanning and multifocal techniques.

## Figures and Tables

**Table 1:** Modeling parameters and variables.

Model Param.	Description	Values and Units	Ref.
*F_a_*	Fraction of absorbed photons that do not result in two-photon emission	—	—
*N_a_*	Number of absorbed photons	—	—
*N_τ_*	Total number of photons	—	—
*N_1λ_*	Number of photons absorbed due to one-photon absorption of the medium	—	—
*N_2λ_-N_em_*	Number of absorbed photons that do not result in two-photon emission	—	—
*µ_a_*	Absorption coefficient	1 cm^−1^	[25,26,27]
*l*	Propagation length (two Rayleigh lengths)	5 µm	—
*I*	Peak power density	W·cm^−2^	—
*S*	Coherence parameter	—	[28]
*δ_2λ_*	Two-photon cross-section at 960 nm	31 × 10^−50^ cm^4^·s^−1^·molec. ^−1^·photon^−1^	[11]
*C*	Typical concentration of fluorophores	6 × 10^18^ molec.·cm^−3^	[29]
*λ*	Wavelength used for two-photon imaging	960 nm	—
*h*	Planck constant	6.62607015 × 10^−34^ J·s^−1^·photon^−1^	—
*c*	Speed of light in vacuum	299,792,458 m·s^−1^	—
Φ	Quantum efficiency	0.67	[11]
*ε*	Heat source	J·m^−3^	—
*P_A_*	Average laser power	W	—
*V*	Volume of the excitation cylinder	µm^3^	—
*q_r_*	Heat	J	—
*T*	Temperature	K	—
*k*	Specific thermal conductivity of brain tissue	0.5918 W·K^−1^·m^−1^	[30,31]
